# CO_2_‐Assisted Hydrolysis of NaBH_4_: A Multi‐Function Platform for Enhanced Hydrogen Release, Regeneration of NaBH_4_, and CO_2_‐to‐CH_4_ Conversion

**DOI:** 10.1002/advs.75677

**Published:** 2026-05-15

**Authors:** Rui Han, Stella Zhang, Yan Dai, Michael Scott, Brandon He, Sadegh Shabani, Oliver Hutt, Matthew David, Zhenguo Huang

**Affiliations:** ^1^ School of Civil and Environmental Engineering University of Technology Sydney Ultimo New South Wales Australia; ^2^ Boron Molecular Noble Park Victoria Australia; ^3^ GrapheneX Sydney New South Wales Australia

**Keywords:** borohydride, carbon dioxide, hydrogen release, hydrogen, regeneration

## Abstract

Sodium borohydride (NaBH_4_) is a promising hydrogen storage material due to its high hydrogen content and facile high‐purity hydrogen release via hydrolysis. This work reports that carbon dioxide (CO_2_) accelerates the hydrolysis of NaBH_4_ and facilitates the regeneration of NaBH_4_ via ball milling the hydrolytic products with Mg. CO_2_ significantly enhances the hydrogen release by providing protons required during hydrolysis. It also facilitates the formation of borax during hydrolysis which can be used directly as feedstocks to regenerate NaBH_4_ with a high yield of over 76%. Compared to widely used metal‐based catalysts, CO_2_‐assisted hydrolysis offers significant advantages since it eliminates the need for metal catalyst separation from the hydrolytic products, simplifying recovery and enabling more efficient NaBH_4_ regeneration. Moreover, CO_2_ is converted to CH_4_ during the process, achieving both carbon capture and conversion. This multi‐functional approach contributes to the development of a closed‐loop hydrogen storage system, accompanied by an efficient CO_2_ conversion to a power fuel.

## Graphical abstract

CO_2_ significantly accelerates hydrogen release from aqueous NaBH_4_ without metal catalysts, eliminating the need for downstream separation. Ball‐milling the hydrolytic products with Mg regenerates NaBH_4_ with yields exceeding 76% while simultaneously producing methane. This integrated process establishes a closed‐loop hydrogen storage cycle based on NaBH_4_, enabling both efficient H_2_ delivery and concurrent carbon capture and conversion.

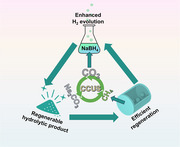

## 1 Introduction

NaBH_4_ has long been considered one of the most promising hydrogen carriers due to its high hydrogen content and its ability to release high‐purity hydrogen on demand through hydrolysis. However, the practical use of NaBH_4_ as a hydrogen carrier is limited by a few challenges. First, the un‐catalysed hydrogen release from neutral NaBH_4_ aqueous solution is slow, limiting its applications where rapid hydrogen demand is needed [1]. For long‐term storage, NaBH_4_ is normally dissolved in caustic solutions to prevent spontaneous hydrogen release. Storage and handling of this corrosive solution require compatible materials which can increase cost. In addition, metal‐based catalysts are needed to accelerate hydrogen evolution, including precious metal catalysts such as Pt [2, 3, 4], Ru [2, 3, 5], Pd [3, 6], and transition metal‐based catalysts [5, 7, 8, 9, 10]. The introduction of these metal catalysts not only increases operating cost, especially in the case of noble metals, but also reduces the net hydrogen capacity of the aqueous system. Furthermore, for practical applications, these catalyst powders need to be efficiently recovered from the hydrolytic products to reduce operating costs and to avoid becoming contaminants in the regenerated NaBH_4_.

Another key challenge in practical applications of NaBH_4_ is the lack of an efficient and cost‐effective regeneration process, ideally using the hydrolytic product, which is normally sodium metaborate. The commercial syntheses of NaBH_4_ are based upon either the Brown‐Schlesinger Process [11] or the Bayer Process [12], both require the use of borax, hydrogen gas, and sodium at elevated temperatures. These methods are energy intensive and need extra caution due to the dangerous nature of the reactants. NaBH_4_ can be regenerated from its hydrolytic product NaBO_2_ via mechanochemical reactions, but this often requires highly reactive and expensive reagents such as MgH_2_ [13, 14, 15]. Zhu et al. reported an efficient regeneration method involving ball milling a mixture of borax decahydrate, Mg and Na_2_CO_3,_ where the borax decahydrate was recovered by treating a hydrolysed NaBH_4_ solution with CO_2_ [16].

**FIGURE 1:**
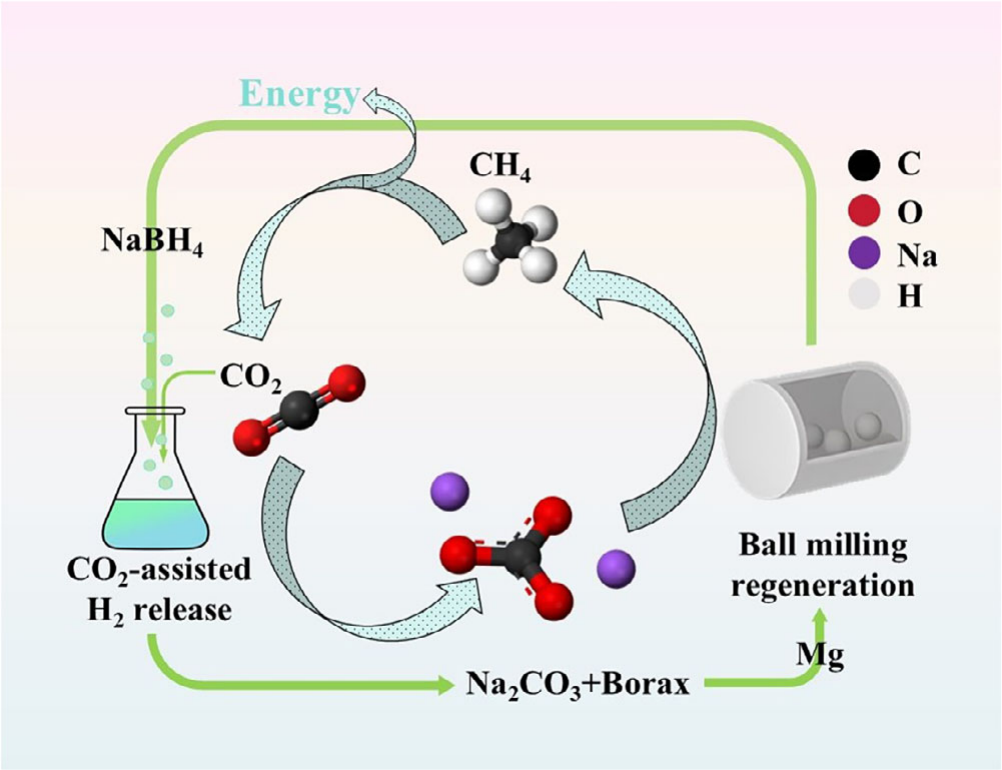
CO_2_‐assisted hydrogen cycle by NaBH_4_ features enhanced hydrogen release, facile hydrolytic products recovery, efficient regeneration of NaBH_4_, and CO_2_‐to‐CH_4_ conversion.

In this study, we present an effective approach to accelerating the hydrolysis of NaBH_4_ using CO_2_. We systematically investigated the effects of CO_2_ on the entire NaBH_4_ hydrogen energy system including H_2_ release rate, hydrolytic products recovery, regeneration of NaBH_4_, and conversion of CO_2_. The use of CO_2_ in the hydrolysis increases the hydrogen production rate by more than 700% compared with unmodified NaBH_4_ aqueous solution. By ball milling the reisolated hydrolytic products with Mg, NaBH_4_ can be regenerated with a yield of up to 76.3%. At the same time, CH_4_ is produced during ball milling, which represents a potential carbon capture, utilisation and storage (CCUS) (Figure 1).

## 2 Results and Discussion

**FIGURE 2:**
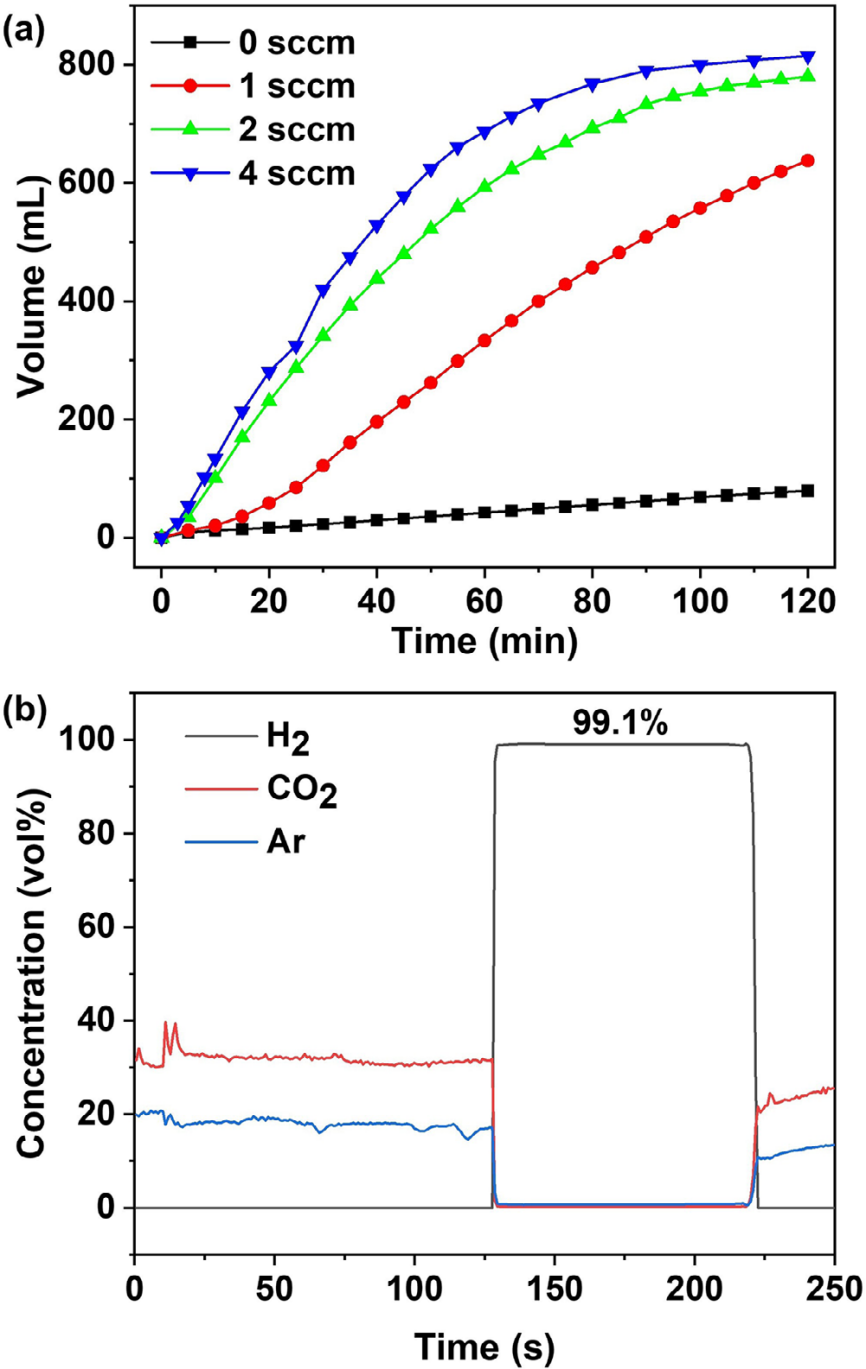
Hydrogen evolution rates for the unmodified NaBH_4_ aqueous solution (0 sccm), CO_2_‐assisted hydrolysis (1 sccm, 2 sccm, and 4 sccm); (b) MS analysis of the gas collected for the 4 sccm.

The hydrolysis of unmodified NaBH_4_ in aqueous solutions (control solution), i.e. NaBH_4_ dissolved in deionised water, has a very low hydrogen evolution rate, while a slow injection of CO_2_ significantly accelerated the reaction. A diagram depicting the experimental setup is shown in Figure S1. Figure 2 shows the hydrogen evolution rates of aqueous solutions of NaBH_4_ with varying CO_2_ addition rates of 0 to 4 sccm over 120 min. By introducing just 1 sccm of CO_2_ to the reaction, the average H_2_ evolution rate became 8 times faster than that of the control solution. When the CO_2_ flow rate is doubled from 1 to 2 sccm, the H_2_ evolution rate increased by 22%. Further increasing the CO_2_ flow rate from 2 to 4 sccm resulted in a slight increase in hydrogen evolution rate of 4.5%. Mass spectroscopy (MS) analysis showed that the CO_2_ concentration in the gas collected was less than 0.30 vol% for 4 sccm (Figure 2b), 0.17 vol% for the 2 sccm and 0.08 vol% for 1 sccm (Figure S2), demonstrating the effectiveness of a simple NaOH solution‐based trap in removing CO_2_. A better design of CO_2_ trap should further improve the purity of the H_2_ gas. As the performance enhancement for the 4 sccm is only marginal compared with the 2 sccm and more residual CO_2_ was found in the H_2_ stream, we did not further increase the CO_2_ flow rate, and the following discussion is mainly focused on 1 and 2 sccm.

**FIGURE 3:**
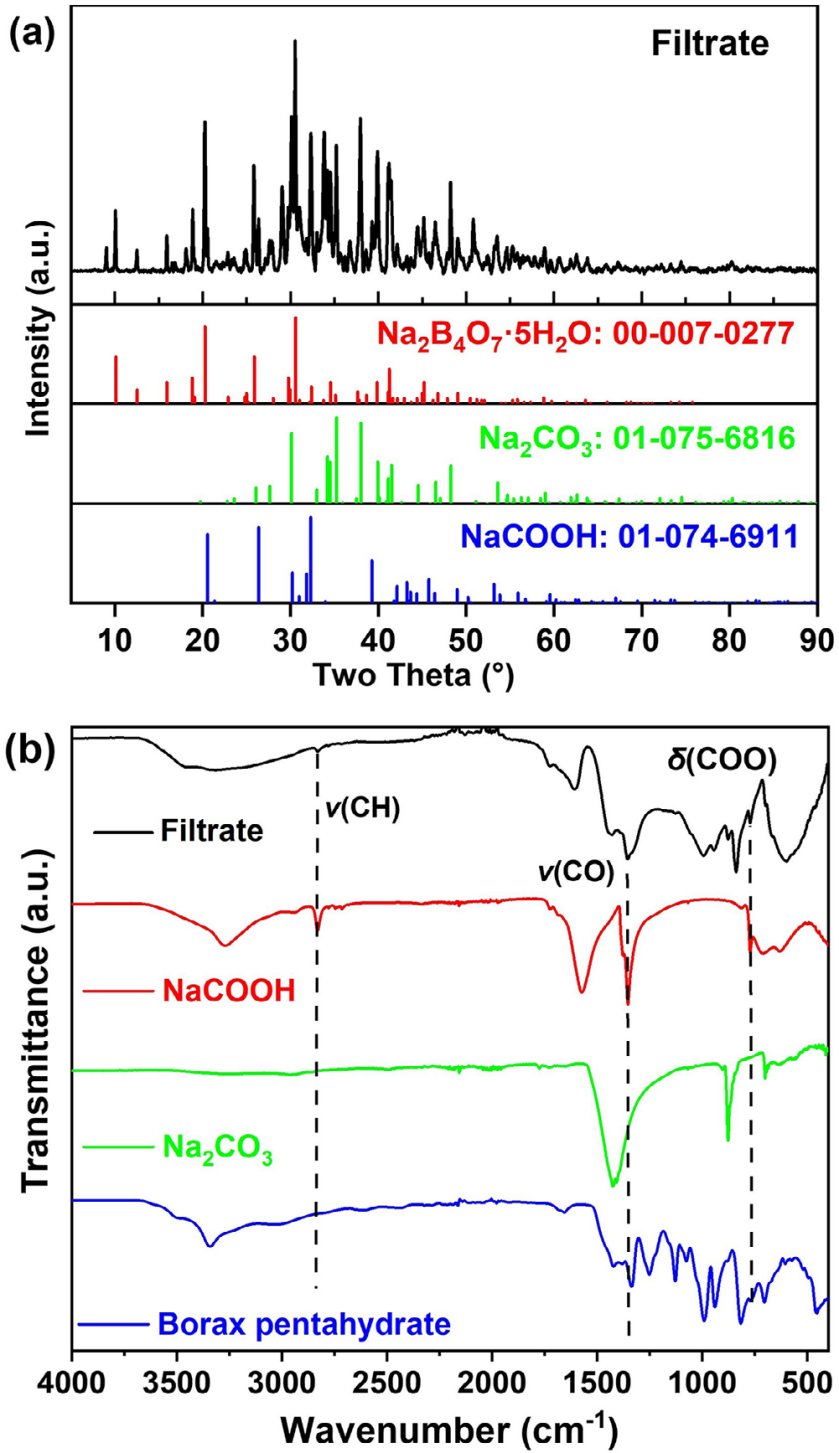
Characterisation of hydrolytic products obtained from the 10 sccm reaction: (a) XRD pattern and (b) FTIR spectrum of the dried products from the filtrate, which consists of borax pentahydrate, Na_2_CO_3_ and NaCOOH.

After the CO_2_‐assisted hydrolysis reactions were completed, the solutions were cooled from 25°C to 0°C and stirred for 30 min, the resulting precipitate was isolated by filtration, as white crystals. Additional powders were recovered after drying the filtrate. Characterisation was carried out on both the filtered solid and the dried hydrolytic products from the filtrate. XRD patterns and FTIR spectra were collected to analyse these solids. The XRD pattern and FTIR spectrum of filtered solid are consistent with borax decahydrate (Figure S4). Most of the borax decahydrate can be easily separated out due to its relatively low solubility at 0°C (1.99 g/100 mL). TGA results show that the air‐dried borax exhibits the same mass loss and heat flow profile as the commercial one (Figure S5), demonstrating the air‐drying process is efficient to remove moisture. The XRD pattern (Figure 3a) of the dried products from the filtrate (denoted Filtrate) matches well with the combined diffraction patterns of borax pentahydrate (PDF 00‐007‐0277), sodium carbonate (PDF 01‐075‐6816), and sodium formate (PDF 01‐074‐6911). The borax pentahydrate in the Filtrate results from the soluble borax being dried under dynamic vacuum at an elevated temperature [17, 18]. FTIR analysis was conducted to validate the existence of these three compounds. As shown in Figure 3b, the FTIR spectrum of the Filtrate contains IR bands that can be attributed to [B_4_O_5_(OH)_4_]^2^^−^ from borax pentahydrate and CO_3_^2−^ (1420, 875 cm^−1^) from Na_2_CO_3_. The FTIR spectrum also shows the stretching vibrations of C–H and C–O at 2830 cm^−1^ and 1350 cm^−1^, respectively, as well as the bending vibration of COO at 765 cm^−1^, which are unique to COOH^−^, confirming the presence of NaCOOH in the filtrate. The formation of NaCOOH is due to the redox reaction between CO_2_ and BH_4_^−^ in aqueous solution, which has been previously reported [19]. ^13^C NMR (Figure S6) for the hydrolytic solution confirmed formate was the only carbon product of the redox reaction between CO_2_ and BH_4_^−^. Combining the results for the filtered solid residue, it can be concluded that the hydrolytic products of CO_2_‐assisted NaBH_4_ hydrolysis are borax, Na_2_CO_3_ and NaCOOH.

The co‐existence of carbonate and formate suggests two reaction pathways for the CO_2_‐assisted hydrolysis of NaBH_4_. In the reaction pathway (*P#1*) that produces carbonate, there is no redox reaction between CO_2_ and NaBH_4_. The *P#1* reaction can be illustrated as follows (Equation 1):

(1) $$\begin{array}{ccc} & & 4{\mathrm{NaBH}}_{4}+16H_{2}O+{\mathrm{CO}}_{2}+2H_{2}O\\ & & \;\to \;{\mathrm{Na}}_{2}B_{4}O_{7}\cdot 10H_{2}O+{\mathrm{Na}}_{2}{\mathrm{CO}}_{3}+16H_{2} \end{array}$$

The amount of H_2_ generated this pathway is the same as that of the unmodified NaBH_4_ aqueous solution, i.e., one equivalent of NaBH_4_ producing four equivalents of H_2_. In the second reaction pathway (*P#2*), BH_4_^−^ and CO_2_ in aqueous solution underwent a redox reaction resulting in the reduction of CO_2_ to COOH^−^ [20]. Considering that the hydrolytic products contain borax, NaCOOH, the reaction for *P#2* can be assumed as follows (Equation 2):

(2) $$\begin{array}{ccc} & & 4{\mathrm{NaBH}}_{4}+2{\mathrm{CO}}_{2}+17H_{2}O\\ & & \;\to \;{\mathrm{Na}}_{2}B_{4}O_{7}\cdot 10H_{2}O+2\mathrm{NaCOOH}+14H_{2} \end{array}$$

In order to quantify the amount of NaCOOH, ion chromatography was conducted. The mass percentage of NaCOOH in the Filtrate was determined to be 7.43 wt.%. As the total molar amounts of boron and sodium remain the same before and after the reaction, the content of borax and sodium carbonate can be calculated accordingly (Table S2). According to the calculation, the ratio of n(borax): n(Na_2_CO_3_): n(NaCOOH) is 6.14: 5.63: 1, which indicates the percent of NaBH_4_ consumed via *P#1* is 91.8% (Equation S7). Compared with *P#1*, 2 equiv. H^−^ were used to reduce 2 equiv. CO_2_, resulting in 2 equiv. less H_2_ generated for every 4 equiv. of NaBH_4_. Considering the amount of NaCOOH formed, there is one H^−^ to reduce CO_2_ in every 98.4 H^−^, translating to a 1% hydrogen loss compared with hydrolysis of the unmodified NaBH_4_. The volume of H_2_ collected experimentally for the CO_2_‐assisted hydrolysis was close to the theoretical total H_2_ yield for both pathways. To conclude, our analysis shows that the majority of NaBH_4_ is consumed via *P#1* with significantly enhanced hydrogen evolution rate and minimal hydrogen loss.

Injecting CO_2_ to the NaBH_4_ hydrolysis significantly improves the hydrogen evolution, and it is important to understand the mechanism. CO_2_ dissolves in water forming carbonic acid (Equation 3) which makes the reaction environment more acidic and thus there are more protons to react with NaBH_4_, compared with unmodified NaBH_4_ aqueous solution (Equation 4). The pH values of CO_2_‐assisted NaBH_4_ hydrolysis solution were measured every 10 min throughout the reaction (Figure S7). The initial pH values were 9.9 and 10.0 for the 1 and 2 sccm experiments, respectively. For 1 sccm, the pH value increased slightly to 10.3 before decreased to 9.8 and remained stable throughout the reaction. For the 2 sccm experiment, the pH value decreased to 9.6 and remained stable during most of the measurement and dropped to 9.5 nearing the end. With the injection of CO_2_, the hydrogen evolution rate (i.e., the slope of the curve) increased drastically compared with the hydrolysis of unmodified NaBH_4_. Notably, from the 25^th^ to the 50^th^ min, the hydrogen evolution rates and the pH values remained relatively constant (dashed box in Figure S7). Control experiments with representative hydrolytic products (Na_2_CO_3_, NaCOOH, and borax) show that these species have limited or inconsistent effects on hydrogen evolution, whereas different buffer solutions at similar pH consistently accelerate the reaction (Figure S8). This indicates that product formation alone does not account for the observed kinetics. Instead, even modest pH reductions induced by CO_2_, through increased proton availability, are the dominant factor driving the enhanced hydrogen evolution rate.

(3) $$\begin{array}{c} {\mathrm{CO}}_{2}+H_{2}O\;⇌\;H_{2}{\mathrm{CO}}_{3} \end{array}$$

(4) $$\begin{array}{c} {\mathrm{NaBH}}_{4}+2H_{2}O\;\to \;{\mathrm{NaBO}}_{2}\cdot 2H_{2}O+4H_{2} \end{array}$$

**FIGURE 4:**
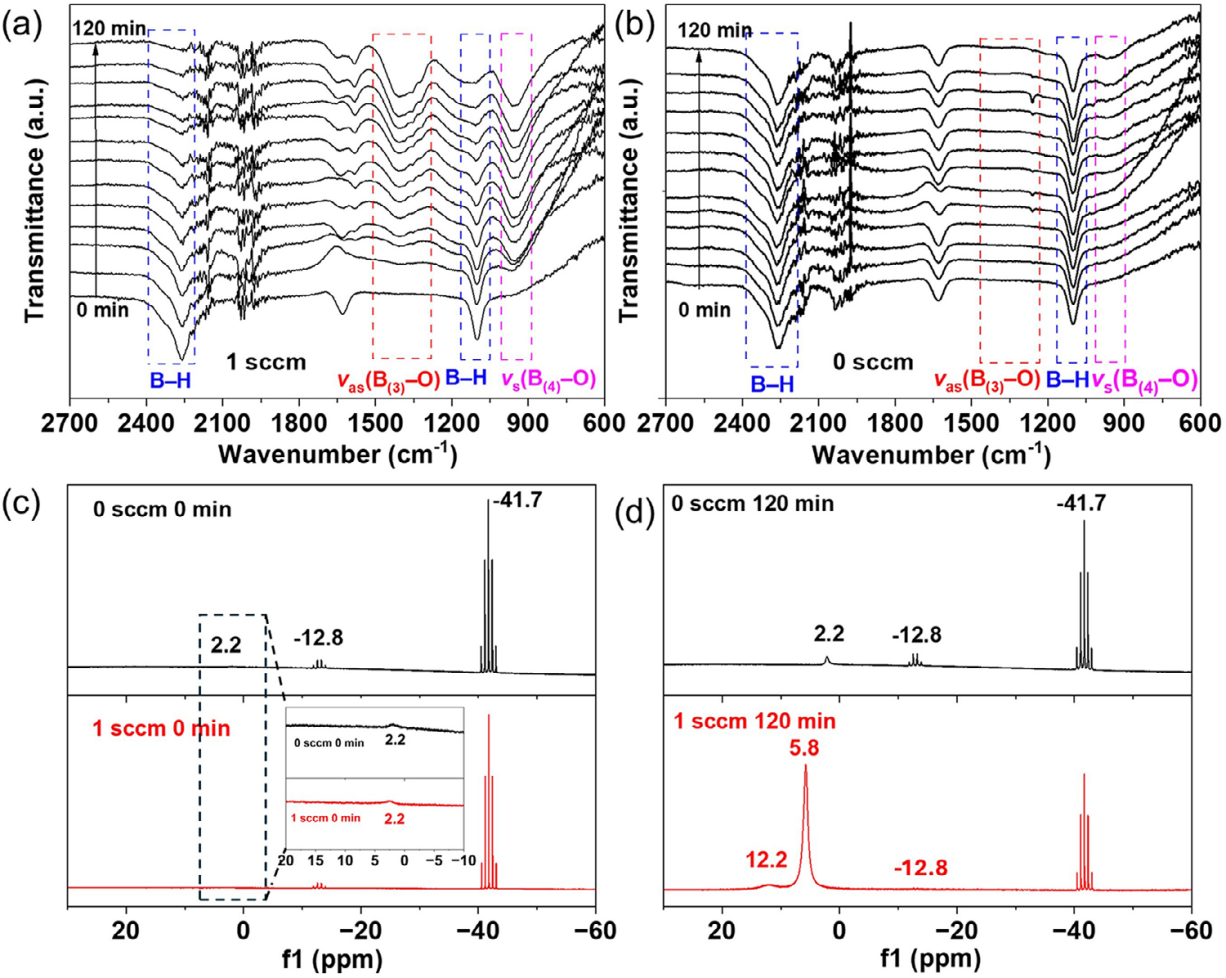
FTIR spectra of the NaBH_4_ aqueous solution over 120 min for (a) 1 sccm and (b) 0 sccm. Measurements are taken every 10 min. ^11^B NMR spectra (c) at 0 min for both 0 sccm and 1 sccm, and (d) at 120 min for both 0 sccm and 1 sccm.

The hydrolytic product of unmodified NaBH_4_ is sodium metaborate NaB(OH)_4_, which contains four‐coordinate boron in B(OH)_4_^−^. Borax is a polyborate and its [B_4_O_5_(OH)_4_]^2^^−^ anion contains two four‐coordinate boron centres and two three‐coordinate boron centres. The [B_4_O_5_(OH)_4_]^2^^−^ anion is formed by condensation of B(OH)_3_ and B(OH)_4_^−^ forming a B–O–B bond and H_2_O (Equation 5). At low concentrations, polyborate tends to hydrolyse and revert to B(OH)_3_ and B(OH)_4_^−^. [18] From regeneration perspectives, borax is more ideal than metaborate. FTIR and ^11^B NMR (Figure 4) were conducted for both 0 and 1 sccm reactions to investigate the reason why borax was formed during the CO_2_‐assisted hydrolysis. IR bands were detected at 2100–2400 cm^−1^ and 1100 cm^−1^ at the beginning of both reactions, which are associated with B–H bond in unreacted NaBH_4_ [21]. For the 1 sccm, IR bands at 960 cm^−1^ and 1,400 cm^−1^ appeared after 10 min, assigned to *v*_s_(B_(4)_–O) and *ν_as_* (B_(3)_–O), respectively [22]. As the experiments progressed, IR bands associated with B–H became weaker due to the gradual depletion of NaBH_4_, and the IR bands for *ν_as_* (B_(3)_–O) and *v*_s_(B_(4)_–O) became stronger. On the other hand, there is no obvious IR bands associated with three‐coordinate boron species for the 0 sccm throughout the reaction. The IR band for *v*_s_(B_(4)_–O) emerged after 60 min for 0 sccm, which is attributed to the formation of NaB(OH)_4_ as a result of slow hydrolysis of NaBH_4_.

The ^11^B NMR spectra (Figure 4c,d) at 0 min for both 0 sccm and 1sccm, showed the quintet centred at ‐41.7 ppm associated with BH_4_^−^, a weak quartet centred at ‐12.8 ppm associated with the partially dehydrogenated BH_3_(OH)^−^ anion [23], and a very weak peak at 2.2 ppm associated with the hydrolytic product, i.e., B(OH)_4_^−^. After 120 min, the 0 sccm showed the same hydrolytic product at 2.2 ppm associated with B(OH)_4_^−^, whereas the 1 sccm showed two different peaks with the prominent peak representing the B(OH)_3_–B(OH)_4_^−^ equilibrium at 5.8 ppm and a broad peak with lower intensity representing polyborate at 12.2 ppm. In aqueous solution, B(OH)_3_ and B(OH)_4_^−^ exist in a pH‐dependent equilibrium (Equation 6). This equilibrium appears as a single ^11^B NMR resonance with increasing B(OH)_3_ content at lower pH values. While at higher pH values, the concentration of B(OH)_4_^−^ increases [24]. Both FTIR and ^11^B NMR results show that CO_2_ injection leads to the formation of more B(OH)_3_ during hydrolysis. The increased B(OH)_3_ concentration facilitates the reaction between B(OH)_3_ and B(OH)_4_^−^ to produce polyborate [18, 24, 25].

(5) $$\begin{array}{c} 2B\left(\mathrm{OH}\right)4^{-}+2B{\left(\mathrm{OH}\right)}_{3}\;⇌\;{\left[B_{4}O_{5}{\left(\mathrm{OH}\right)}_{4}\right]}^{2-}+5H_{2}O \end{array}$$

(6) $$\begin{array}{c} B\left(\mathrm{OH}\right)4^{-}+H^{+}⇌\;B{\left(\mathrm{OH}\right)}_{3}+H_{2}O \end{array}$$

In a recent study, borax decahydrate, Na_2_CO_3_ and Mg were used as the starting materials for the mechanochemical synthesis of NaBH_4_ [16]. Borax decahydrate was produced by reacting CO_2_ with sodium metaborate solution. The hydrogen source comes from the structural water in borax decahydrate, avoiding the use of hazardous and expensive metal hydrides. For the regeneration of NaBH_4_, we followed the same procedure and used the borax decahydrate isolated from the hydrolytic solution as the boron and hydrogen source without further treatment. The overall reaction is expressed as follows (Equation 7).

(7) $$\begin{array}{ccc} & & {\mathrm{Na}}_{2}B_{4}O_{7}\cdot 10H_{2}O+{\mathrm{Na}}_{2}{\mathrm{CO}}_{3}+20\;\mathrm{Mg}\\ & & \;\to \;4{\mathrm{NaBH}}_{4}+20\mathrm{MgO}+{\mathrm{CH}}_{4} \end{array}$$

At a certain molar ratio (Mg: Na_2_CO_3_: borax decahydrate = 22:1:1), a NaBH_4_ yield of 76.3% was achieved on a planetary ball mill after 80 h of milling at 400 CPM. Figure S9 shows the milling time and speed's effects on the NaBH_4_ yield. A 69.5% NaBH_4_ yield was achieved on a high energy ball mill after 20 h at 1200 CPM. The high energy ball mill is more efficient in producing NaBH_4_ due to the higher local impact between the grinding balls and the reagents. Interested readers can refer to Zhu et al.’s article on different ball mill parameters’ effects on the yield [15]. FTIR and XRD analyses (Figure S10) demonstrate that high‐quality NaBH_4_ was obtained by ball milling Mg with Na_2_CO_3_ and the recovered borax decahydrate from the CO_2_‐assisted NaBH_4_ hydrolysis. High specific energy consumption was observed for NaBH_4_ regeneration. This is primarily due to the small‐scale experimental conditions, where most energy is expended in rotating grinding jars with masses far exceeding that of the feedstock. The energy consumption is expected to decrease substantially upon scale‐up and process optimisation.

Methane (CH_4_) was also detected in the grinding jar, as evidenced by the gas analysis via MS (Figure S11). CH_4_ is produced as a result of the reduction of Na_2_CO_3_ isolated from the hydrolytic products, and carbon in Na_2_CO_3_ ultimately comes from CO_2_ injected during the hydrolysis. The carbon conversion rate by ball mill is defined as the percentage of carbon in Na_2_CO_3_ converted to CH_4_ during NaBH_4_ regeneration (Equation S13). The overall carbon conversion rate is defined as the fraction of total CO_2_ introduced into the system that is converted to CH_4_ (Equation S14). The carbon conversion rate by ball mill reflects the efficiency of ball mill regeneration in converting Na_2_CO_3_ to CH_4_, whereas the overall carbon conversion rate represents the efficiency of the entire hydrogen loop of the CO_2_‐assisted hydrolysis in converting CO_2_ to CH_4_.

An average carbon conversion rate by ball mill of 44.5% was recorded across the measurements of two different samples with an average NaBH_4_ yield of 51.2%, giving an overall carbon conversion rate of 13.7% (Figure S11). The remaining CO_2_ is primarily captured as Na_2_CO_3_ in the NaOH trap. Further optimisation of the experimental conditions is expected to improve the overall carbon conversion rate. Tracing the carbon footprint from CO_2_ to carbonate and to CH_4_, the CO_2_‐assisted hydrogen release of NaBH_4_ and its regeneration is accompanied by a green process of CCUS, which brings additional value when using NaBH_4_ as a hydrogen carrier.

## 3 Conclusion

In this study, CO_2_ was found to enhance the NaBH_4_ hydrogen release via hydrolysis. Injecting CO_2_ shifts the pH of the solution increasing the concentration of protons for the hydrolysis of NaBH_4_, and it also facilitates the formation of borax during hydrolysis. The use of CO_2_ circumvents the separation required in conventional NaBH_4_ hydrolysis facilitated by metal‐based catalysts. Additionally, borax decahydrate, the primary hydrolytic product, can be efficiently isolated via filtration and drying in air, offering an energy‐efficient pathway for recovery. For NaBH_4_ regeneration, high purity NaBH_4_ was obtained via a facile mechanochemical process using the recovered borax decahydrate as both the boron and hydrogen sources, achieving a yield of 76.3%. During the regeneration, the C–O and C = O in the carbonate are converted to C–H in CH_4_, which represents a potential pathway for CCUS. Together, these features make the CO_2_‐assisted NaBH_4_ system a cost‐effective, sustainable, and closed‐loop solution for hydrogen storage and release.

## Conflicts of Interest

The authors declare no conflict of interest.

## Supporting information




**Supporting File**: advs75677‐sup‐0001‐SuppMat.docx.

## Data Availability

The data that support the findings of this study are available from the corresponding author upon reasonable request.
