# Supplementary material for: CO2‐Assisted Hydrolysis of NaBH4: A Multi‐Function Platform for Enhanced Hydrogen Release, Regeneration of NaBH4, and CO2‐to‐CH4 Conversion
Source: Adv Sci (Weinh). 2026 May 15;13(44):e75677. doi: 10.1002/advs.75677 (PMC13335977; doi:10.1002/advs.75677)
Supplement: Supplementary file 1 [file ADVS-13-e75677-s001.docx]

Supporting Information

CO_2_-Assisted Hydrolysis of NaBH_4_: A Multi-Function Platform for Enhanced Hydrogen Release, Regeneration of NaBH_4_, and CO_2_-to-CH_4_ Conversion

Rui Han, Stella Zhang, Yan Dai, Michael Scott, Brandon He, Sadegh Shabani, Oliver Hutt, Matthew David and Zhenguo Huang*

Experimental

Reagents and materials

NaBH_4_ (≥98%), Mg (≥99%), Na_2_CO_3_ (>99%), ethylene diamine (>99%), borax decahydrate (>99%), NaOH (≥ 97%) were purchased from Sigma Aldrich and used without further purification. CO_2_ (grade 2.5) was purchased from Coregas. The calibration gas mixture was purchased from CAC Gas and Instrumentation.

CO_2_-assisted hydrogen release via NaBH_4_ hydrolysis

The experimental setup is shown in Figure S1. 300 mg NaBH_4_ was first loaded into a 50 mL round bottom flask. 20 mL of deionised water was then injected into the flask via a syringe. CO_2_ was introduced into the solution via a long needle, and the flow rate was controlled by a mass flow controller (Alicat MC-200SCCM). The mass flow rate ranges from 1 sccm to 4 sccm. The reaction was stirred and the gas outlet of the flask was connected to a CO_2_ trap filled with 60 mL 2M NaOH. A burette was connected to the other side of the CO_2_ trap for hydrogen collection. The volume of the gas in the burette was recorded every 5 minutes for 120 minutes.

**Figure S1**. The illustration of experimental setup of the CO_2_-assisted hydrolysis of NaBH_4_

Hydrolytic products recovery and characterisation

For hydrolytic products recovery, the CO_2_-assisted hydrogen release was done on a 10x scale. In a typical experiment, 3 g NaBH_4_ was dissolved in 50 mL of DI water with CO_2_ flow rate ranging from 10 sccm to 40 sccm for 120 minutes. After the reaction was complete, the solution was cooled to 0 °C for 30 minutes and white precipitation was filtered out using a filter paper. The filtered solid was left in air overnight for drying. The filtrate was dried using a rotary evaporator at a vacuum level of 40 mbar and a temperature of 40 °C. X-ray diffraction (Bruker D8 Discover), Fourier Transform Infrared spectroscopy (Nicolet Rapt FTIR Microscope), thermogravimetric analysis (SETARAM Thermys One+) and nuclear magnetic resonance (Bruker D400 NMR) spectroscopy analysis were performed to characterise the recovered hydrolytic products. Formate concentration measurements were performed on a Thermo Scientific Dionex ICS-6000 HPIC system equipped with an AS18-Fast-4 µm analytical/guard column set. A KOH EGC-500 cartridge was employed for Eluent generation. Samples were diluted 20 to 100 times with DI water and calibrated using a formate standard with a 10 ppm QC check per batch. Formate concentrations were determined from retention time and peak area.

Regeneration of NaBH_4_

The regeneration of NaBH_4_ was carried out on two different ball mills. On the planetary ball mill (Retsch PM 200), a mixture of 1.120 g recovered borax decahydrate, 1.570 g of Mg and 0.310 g Na_2_CO_3_ was milled for 80 hours at 400 cycles per minute (CPM). On the high-energy ball mill (Across International VQN), a mixture of 0.175 g recovered borax decahydrate, 0.276 g Mg and 0.049 g Na_2_CO_3_ was milled for 20 hours at 1200 CPM. The loading of starting materials was done in an argon filled glovebox. A custom-made ball jar with valved gas outlet was used for the regeneration of NaBH_4_ and collecting the gases. The yield was determined from iodometry.^[1]^

Gas analysis

The gas generated by the CO_2_-assisted hydrolysis of NaBH_4_ was collected using a gas sampling bag with valves. The gas sampling bag was purged by vacuuming and filling with argon three times. The gas collected in the gas sampling bag was fed into the MS analyser with a background flow of Ar. The multi-concentration analyses for the 1 sccm and 2 sccm are shown in Figure S2.

**Figure S2.** Gas analysis for (a) 1 sccm and (b) 2 sccm CO_2_-assisted hydrolysis of NaBH_4_.

Gas analysis on the gases formed during the ball milling was conducted on a MS analyser (ThermoStar QMG220). The gas inlet of the MS was connected to the gas outlet of the ball mill jar.

**Statistical Analysis**

Hydrogen evolution data were recorded as a function of time and analysed directly. Hydrogen evolution experiments were conducted with three replicates. Data are presented as mean. Initial hydrogen evolution rates were determined from the linear region at early reaction times. No data transformation or normalisation was applied. Outliers were not excluded unless a clear experimental error (e.g., leakage or mass flowmeter malfunction) was identified. No formal statistical hypothesis testing was performed. Comparisons between different experimental conditions are based on the observed trends and reproducibility across independent measurements. All data processing and analysis were performed using OriginPro.

The impact of CO_2_ inject rate on reactions

To collect enough hydrolytic products for the regeneration of NaBH_4_, the reactions were conducted at a 10 times scale, i.e., both the flow rate and the NaBH_4_ mass increasing by 10 times. It is well known that structures of borates in an aqueous solution are largely dependent on the pH value.^[2]^ The pH values of the reactions with CO_2_ flow rates of 1 sccm, 2 sccm, 10 sccm, and 20 sccm, were between 9.8-10.3 at the beginning and between 9.5-9.8 at the end, whereas the pH values of unmodified NaBH_4_ hydrolysis started from 10.0 and slightly increased to 10.2 at 120 min (Table S1). The semi-*in-situ* FTIR results of the liquid intermediates (Figure S3) reveal very similar IR spectra for all CO_2_-assisted reactions. At the beginning of the CO_2_-assisted reactions, the IR bands associated with B–H bond (1100 cm^-1^, 2100–2400 cm^-1^) were at their strongest due to the unreacted NaBH_4_ and gradually became weaker due to NaBH_4_ consumption. Both *v*_as_(B_(3)_–O) (1400 cm^-1^) and *v*_s_(B_(4)_–O) (960 cm^-1^) appeared within 10 minutes of the reaction and gradually became stronger. The starting and endpoint pH values of the reactions are listed in Table S1. These indicate that the 10 sccm and 1 sccm reactions take place in very similar ways. The same applies for the 20 sccm and 2 sccm reactions.

**Table S1.** The pH values of the CO_2_-assisted hydrolysis solutions.

| Reactions | pH (start) | pH (finish) |
| --- | --- | --- |
| 0 sccm | 10.0 | 10.2 |
| 1 sccm | 10.3 | 9.8 |
| 10 sccm | 10.0 | 9.6 |
| 2 sccm | 10.0 | 9.5 |
| 20 sccm | 9.8 | 9.7 |

**Figure S3.** FTIR spectra of the CO_2_-assisted hydrolysis solutions from 0 to 120 min: (a) 1 sccm; (b) 10 sccm; (c) 2 sccm and (d) 20 sccm.

**Characterisation of the filtered solid**

Figure S4 shows XRD and FTIR analyses for the filtered solid. The XRD pattern can be indexed to borax decahydrate (PDF-04-012-8356). IR spectrum shows *ν_as_* (B_(3)_–O) at 1419 cm^-1^ and 1357 cm^-1^, *δ* (B–O(–H)) at 1150 cm^-1^, *ν_as_* (B_(4)_–O) at 1071 cm^-1^, 1035 cm^-1^ and 997 cm^-1^, and *ν*(O–H) from 3200 cm^-1^ to 3600 cm^-1^, which are characteristic of [B_4_O_5_(OH)_4_]^2-^.^[4]^ These results match those of commercial borax decahydrate.

Figure S4. Characterisation of hydrolytic products obtained from the 10 sccm reaction: (a) XRD patterns and (b) FTIR spectra of the isolated solid precipitate and commercial borax decahydrate.

TGA analysis for the air-dried solid borax decahydrate

**Figure S5.** TGA curves of (a) air-dried filtered solid and (b) commercial borax decahydrate. The highlighted mass loss corresponds to the initial 5 structural water loss from borax decahydrate when heated.

^13^C NMR for the hydrolytic solution

^13^C NMR has been conducted for the hydrolytic solution to investigate if CO_2_ was reduced to other carbon species (Figure S6). D_2_O with 3 wt% 3-(trimethylsilyl)propionic-2,2,3,3-d4 acid sodium salt was added to the hydrolytic solution with a volume ratio of 1:1. Chemical shifts were referenced to 3-(trimethylsilyl)propionic-2,2,3,3-d₄ acid, set at 0 ppm. As shown in Figure S5, there are two peaks at 173.9 ppm and 163.7 ppm,^[3]^ which correspond to formate and bicarbonate, respectively. No other carbon species are found in the NMR spectrum.

**Figure S6.** ^13^C NMR for the hydrolytic solution.

Hydrolytic products quantification

To collect enough hydrolytic products, hydrogen evolution was carried out at a larger scale. Typically, 3 g NaBH_4_ (98% purity) was dissolved in 50 mL of water, and 10 sccm of CO_2_ was injected into the reaction for 120 min. After the hydrolysis was finished, the solution was cooled to 0 °C for 30 minutes and white precipitation was filtered out using a filter paper. The filtrate was dried using a rotary evaporator at a vacuum level of 40 mbar and a temperature of 40 °C. After drying in air, the filtered solid was weighed 6.3 g and the dried products from the filtrate weighed 2.9 g. The NaBH_4_ contains 77.8 mmol of boron and sodium. As the only boron-containing product is borax (including both Na_2_B_4_O_7_∙10H_2_O in the filtered solid and the Na_2_B_4_O_7_∙5H_2_O in the dried by-products from the filtrate), its amount of substance can be calculated according to Eq. S1 and Eq. S2 below.

*n*(Na_total_) = *n*(B_total_) = 3 g x 98%/37.8 g mol^-1^ x 1000 = 77.78 mmol (S1)

*n*(borax_total_) = *n*(B_total_)/4 = 77.78 mmol/4 = 19.45 mmol (S2)

The filtered solid contains only borax decahydrate, and its amount of substance can be obtained from the mass. The *n*(borax pentahydrate) can be therefore obtained from subtracting *n*(borax decahydrate) from *n*(borax_total_), shown in Eq. S3 and Eq. S4.

*n*(borax decahydrate) = 6.3 g/381.4 g mol^-1^ = 16.52 mmol (S3)

*n*(borax pentahydrate) = *n*(borax_total_) - *n*(borax decahydrate) = 19.45 mmol – 16.52 mmol = 2.93 mmol (S4)

The amount of sodium before and after the reaction should also remain the same. After the reaction, the total sodium is the sum of sodium from borax, NaCOOH and Na_2_CO_3_. The amount of NaCOOH can be calculated according to ion chromatography results (Eq. S5). And the amount of Na_2_CO_3_ can be calculated according to Eq. S6.

*n*(NaCOOH) = $\frac{\text{2.9 g × 7.43 wt\% }}{\text{68 g }\text{mol}^{\text{-1}}}\text{ ×1000}\text{ }\text{=}\text{ }\text{3.17 mmol }$(S5)

$n\left( {Na}_{2}{CO}_{3} \right)=\frac{\text{1}}{\text{2}}\text{ × }\left[ \left( \text{n}\text{(}\text{Na}_{\text{total}}\text{) - }\text{n}\text{(NaCOOH)}\text{ }\text{-}\text{ }\text{2}\text{ }\text{×}\text{ }\text{n}\text{(borax} \right) \right]$ = 17.86 mmol (S6)

The composition of the hydrolytic product is summarised in Table S2. The percent of NaBH_4_ consumed via reaction pathway *P#1*(denoted *S_P#1_*) can be calculated from the amounts of Na_2_CO_3_ and NaCOOH (Eq. S7).

*S_P#1_* = $\frac{\text{4 ×}\text{ }\text{n}\text{(}\text{Na}_{\text{2}}\text{CO}_{\text{3}}\text{)}}{\text{4 ×}\text{ }\text{n}\text{(}\text{Na}_{\text{2}}\text{CO}_{\text{3}}\text{)}\text{ + }\text{2 ×}\text{ }\text{n}\text{(NaCOOH)}} \times100\%=91.8\%$ (S7)

**Table S2.** The composition of the hydrolytic product for the 10 sccm reaction.

|  | Na_2_B_4_O_7_∙10H_2_O | Na_2_B_4_O_7_∙5H_2_O | Na_2_CO_3_ | NaCOOH |
| --- | --- | --- | --- | --- |
| Amount of Substance (mmol) | 16.52 | 2.93 | 17.86 | 3.17 |

Theoretical and experimental hydrogen yield

The total H_2_ collected experimentally (1 sccm) is compared with the theoretical yields for both pathways combined. Theoretical yield of H_2_ is calculated according to the equations (Eq. S8) and (Eq. S9). As the measurement was conducted using a burette, the partial pressure of water vapor is considered for measured gas volume (Eq.S10).

$n\left( H_{2} \right)= 4 \times n\left( NaBH_{4} \right)\times\frac{98.6}{98.6+1}$	(S8)

$V=nRT/P$	(S9)

$P_{total}=P_{water} +P_{gas}$	(S10)

CO_2_-assisted experiments (1 sccm) were conducted at a room temperature of 20 ºC using 300 mg NaBH_4_ (98% purity) till completion (gas volume remained stable for 2 hours). The theoretical H_2_ volume can be calculated using the below equation.

*V*_t_ = 300 mg/37.8 mg mmol^-1^ x 98% x 4 x 98.6/(98.6+1) x 24 mL∙mmol^-1^ = 739 mL (S11)

The measured gas volume till completion was averaged at 748 mL across 3 experiments. Considering the water vapour’s partial pressure at 20 °C (17.54 torr) and the H_2_ purity determined by MS analysis, the actual H_2_ volume is 729 mL, which is 1.35% lower than the theoretical yield. This deviation falls within the experimental uncertainty.

**Mechanism discussion**

**Figure S7.** Hydrogen evolution and pH value measurement for the 0 sccm, 1 sccm and 2 sccm reactions.

The introduction of CO_2_ not only alters the pH of the reaction medium but also generates hydrolytic products (borax, Na_2_CO_3_, NaCOOH) distinct from those formed in unmodified NaBH_4_ hydrolysis. To evaluate their contributions to the enhanced hydrogen evolution, we systematically investigated the effects of representative hydrolytic products by adding their aqueous solutions to NaBH_4_ powder and monitoring the hydrogen evolution profiles (Figure S8a).

Borax and Na_2_CO_3_ solutions were prepared at 0.1 M, approximating their concentrations at the completion of CO_2_-assisted hydrolysis. For comparison, NaCOOH was also tested at 0.1 M, which is more than six times its concentration under CO_2_-assisted conditions. As shown in Figure 1a, Na_2_CO_3_ suppresses hydrogen evolution, consistent with its alkaline nature (pH ≈ 10.2). NaCOOH (pH ≈ 7) produces only a slight enhancement, which is negligible under realistic conditions given its much lower concentration. In contrast, borax solution (pH ≈ 9.4) leads to a pronounced increase in hydrogen evolution rate. While this suggests a correlation between borax formation and improved performance, causation cannot be clearly established because formation of borate species is strongly pH-dependent, making it difficult to decouple the effects of borax formation from pH value.

To further isolate the role of pH, a series of buffer solutions (0.5 M) with pH values close to that of the borax solution (pH ≈ 9.4) were prepared, and hydrolysis reactions were conducted under identical conditions. The initial pH values and those after 120 minutes were recorded. As shown in Figure S8b, all buffered systems exhibit significantly enhanced hydrogen evolution rates. Notably, despite having substantially lower borax concentrations than the hydrolysis with the borax solution, hydrolysis with buffered reactions show faster overall kinetics. This indicates that borax formation is unlikely to be the primary driver of the observed enhancement. A trend is also observed: lower initial pH leads to faster initial hydrogen evolution, and lower final pH corresponds to higher average reaction rates.

In summary, the hydrolytic products themselves have a limited impact on hydrogen evolution. The primary factor driving the enhanced performance is the increased proton availability resulting from CO_2_ dissolution, which accelerates NaBH_4_ hydrolysis

**Figure S8.** NaBH_4_ hydrolysis reactions with (a) solutions of hydrolytic products; (b) different buffer solutions.

Milling time and speed’s effects on the NaBH_4_ yield on the planetary ball mill.

On the planetary ball mill, longer milling time leads to higher yield from 0 to 80 hours (Figure S9a). Further increasing the milling time resulted a decrease in yield which might be due to NaBH_4_ decomposition under prolonged milling. Generally, the milling energy is a function of milling speed (cycle per minute or CPM). As shown in Figure S9b, the NaBH_4_ yield was very low (2%) at 300 CPM but increased sharply to 76.3% at 400 CPM and dropped slightly when milling speed is further increased. This is probably due to the milling energy input is too low to drive the reaction at 300 CPM. The milling energy is sufficient to drive the reaction at 400 CPM hence the high yield. Further increase in the CPM may lead to similar effects of prolonged milling or the milling speed approaching the critical speed where the impact between grinding balls and feed materials is compromised.

**Figure S9.** NaBH_4_ yield for planetary ball mill (a) under different milling time at 400 CPM; (b) under different milling speed for 80 hours. Starting materials ratio (Mg: Borax: Na_2_CO_3_ = 22:1:1), total materials loading (3 g) and ball charge (33:1) were fixed.

Characterisation of the regenerated NaBH_4_

FTIR spectrum of the isolated NaBH_4_ from regeneration matches that of commercial NaBH_4_ showing the B–H bending and stretching at 1100 cm^-1^ and 2200–2400 cm^-1^ (Figure S10a). The XRD pattern (Figure S10b) also matches well with the reference (PDF 00-009-0386) and the commercial NaBH_4_.

**Figure S10.** The characterisation of regenerated NaBH_4_ in comparison to commercial product: (a) FTIR spectra and (b) XRD patterns.

Energy consumption of the NaBH_4_ regeneration

The energy consumption of per kg of NaBH_4_ produced from our experiments can be calculated as follows.

$E_{per kg NaBH_{4}}= \frac{Energy consumption per batch (kWh)}{Mass of NaBH_{4} produced per batch (g)}\times1000$	(S12)

**Table S3**. Power consumption details of the mechanochemical regeneration of NaBH_4_

|  | Power at operating conditions (W) | Milling time (h) | NaBH_4_ mass per batch (g) | Total energy consumption (kWh/g) |
| --- | --- | --- | --- | --- |
| High energy ball mill | 150 | 20 | 0.2 | 15 |
| Planetary ball mill | 500 | 80 | 0.675 | 59.25 |

The energy consumptions per kg of NaBH_4_ for the high energy ball mill are calculated to be 15,000 kWh and 59,250 kWh for the high energy and planetary ball mill respectively. The energy consumption is very high for our experimental conditions mainly due to the small scale as most of the energy input is used to drive the rotation of grinding jars which is more than 500 times the mass of the feedstock. Scaling up and optimising the production parameters are expected to drastically reduce the energy consumption of this process.

Carbon conversion calculation

The grinding jars for ball mill regeneration are loaded under Ar atmosphere, so the amount of Ar is known. The ball mill reaction is an air-tight process so the amount of Ar should remain unchanged during the reaction. During the ball mill reaction, H_2_ and CH_4_ were generated. The concentration of H_2_, CH_4_ and Ar were determined by the MS analysis. The carbon conversion rate by ball mill was calculated using the equation below.

${C conversion rate}_{ball mill}\text{= }\frac{\frac{\text{n}\text{(Ar)}}{\text{c}\text{(Ar)}}\text{ × }\text{c}\text{(}\text{CH}_{\text{4}}\text{)}}{\text{n}\text{(}\text{Na}_{\text{2}}\text{CO}_{\text{3}}\text{)}}\text{×100\%}$ (S13)

Figure S11 shows the gas analysis for regeneration samples with NaBH_4_ yield of 52.3% and 50.0%, respectively. When the gas product was fed into the MS analyser, the concentration of Ar, H_2_ and CH_4_ gradually increased until reached a plateau while the concentration of N_2_ decreased. The gas concentrations of Figure. S11(b&d) were obtained via averaging the concentrations at the stable plateau.

**Figure S11.** Gas analysis for two different regeneration samples: (a, b) NaBH_4_ yield = 52.3%, C conversion rate by ball mill = 40.0%; (c, d) NaBH_4_ yield = 50.0%, C conversion rate by ball mill = 49.0%.

The overall carbon conversion rate can be calculated from the composition of the hydrolytic product in Table S2, total CO_2_ injection, and the C conversion rate by ball mill.

$\boldsymbol{C conversion rat}\boldsymbol{e}_{\boldsymbol{overall}}\boldsymbol{=}\frac{\boldsymbol{n}\left( \boldsymbol{N}\boldsymbol{a}_{\boldsymbol{2}}\boldsymbol{CO}_{\boldsymbol{3}} \right)\boldsymbol{\times C conversion rat}\boldsymbol{e}_{\boldsymbol{ball mill}}}{\boldsymbol{C}\boldsymbol{O}_{\boldsymbol{2}}\boldsymbol{Mass flow rate \times time\div22.4 mL\cdot mo}\boldsymbol{l}^{\boldsymbol{-1}}}\boldsymbol{\times100\%}$	(S14)

The overall carbon conversion rate is calculated to be 13.7% for the 10 sccm. This figure can be improved by using regeneration samples with higher yield and through optimisation of experimental conditions.

Reference

[1]	D. A. Lyttle, E. H. Jensen, W. A. Struck, Simple Volumetric Assay for Sodium Borohydride*,* *Analytical Chemistry* **24**, (1952): 1843.

[2]	D. M. Schubert, in *Kirk‐Othmer Encyclopedia of Chemical Technology*, (Eds.: C. Ley), John Wiley & Sons, (2011), pp. 19-43.

[3]	A. V. Ellis, M. A. Wilson, Carbon Exchange in Hot Alkaline Degradation of Glucose, The Journal of Organic Chemistry **67**, (2002): 8469.

[4]	L. Jun, X. Shuping, G. Shiyang, FT-IR and Raman spectroscopic study of hydrated borates*,* *Spectrochimica Acta Part A: Molecular and Biomolecular Spectroscopy* **51**, (1995): 519.
